# Incremental Reduction in Risk of Death Associated With Use of Guideline-Recommended Therapies in Patients With Heart Failure: A Nested Case-Control Analysis of IMPROVE HF

**DOI:** 10.1161/JAHA.111.000018

**Published:** 2012-02-20

**Authors:** Gregg C. Fonarow, Nancy M. Albert, Anne B. Curtis, Mihai Gheorghiade, Yang Liu, Mandeep R. Mehra, Christopher M. O'Connor, Dwight Reynolds, Mary N. Walsh, Clyde W. Yancy

**Affiliations:** Ahmanson-UCLA Cardiomyopathy Center, Ronald Reagan UCLA Medical Center, Los Angeles, CA (G.C.F.); Heart and Vascular Institute, Cleveland Clinic Foundation, Cleveland, OH (N.M.A.); Department of Medicine, University at Buffalo, Buffalo, NY (A.B.C.); Center for Cardiovascular Innovation,Northwestern University Feinberg School of Medicine, Chicago, IL (M.G.); Department of Statistics, CRDM, Medtronic, Inc, Mounds View, MN (Y.L.); Division of Cardiology, Harvard Medical School, Boston, MA (M.R.M.); Division of Cardiology, Duke University Medical Center, Durham, NC (C.M.O.); Division of Cardiology, University of Oklahoma Health Sciences Center, Oklahoma City (D.R.); The Care Group, St Vincent Heart Center of Indiana, Indianapolis, IN (M.N.W.; Division of Cardiology, Northwestern University Feinberg School of Medicine, Chicago, IL (C.W.Y.)

**Keywords:** guideline-recommended therapies, heart failure, nested case-control studies, survival benefit

## Abstract

**Background:**

Several therapies are guideline-recommended to reduce mortality in patients with heart failure (HF) and reduced left ventricular ejection fraction, but the incremental clinical effectiveness of these therapies has not been well studied. We aimed to evaluate the individual and incremental benefits of guideline-recommended HF therapies associated with 24-month survival.

**Methods and Results:**

We performed a nested case-control study of HF patients enrolled in IMPROVE HF. Cases were patients who died within 24 months and controls were patients who survived to 24 months, propensity-matched 1:2 for multiple prognostic variables. Logistic regression was performed, and the attributable mortality risk from incomplete application of each evidence-based therapy among eligible patients was calculated. A total of 1376 cases and 2752 matched controls were identified. β-Blocker and cardiac resynchronization therapy were associated with the greatest 24-month survival benefit (adjusted odds ratio for death 0.42, 95% confidence interval (CI), 0.34–0.52; and 0.44, 95% CI, 0.29–0.67, respectively). Angiotensin-converting enzyme inhibitors/angiotensin receptor blockers, implantable cardioverter-defibrillators, anticoagulation for atrial fibrillation, and HF education were also associated with benefit, whereas aldosterone antagonist use was not. Incremental benefits were observed with each successive therapy, plateauing once any 4 to 5 therapies were provided (adjusted odds ratio 0.31, 95% CI, 0.23–0.42 for 5 or more versus 0/1, *P*<0.0001).

**Conclusions:**

Individual, with a single exception, and incremental use of guideline-recommended therapies was associated with survival benefit, with a potential plateau at 4 to 5 therapies. These data provide further rationale to implement guideline-recommended HF therapies in the absence of contraindications to patients with HF and reduced left ventricular ejection fraction. **(*J Am Heart Assoc*. 2012;1:16-26.)**

Clinical trials have established that several therapies improve clinical outcomes for patients with heart failure (HF) and reduced left ventricular ejection fraction (LVEF).^1–4^ Professional society guidelines for HF recommend the application of these evidence-based therapies in eligible patients without contraindications.^1–4^ However, as a result of the design most commonly used in randomized clinical trials, where new therapies are tested on a background of existing guideline-recommended therapies, the relative clinical value of each new guideline-recommended therapy, independent of other therapies, has not been well studied. Consequently, with new evidence, professional society guidelines usually recommend that each new therapy be added to other established therapies.^1–4^ In addition, despite evidence from clinical trials, questions remain about the translation of efficacy and safety findings from randomized clinical trials to real-world effectiveness in clinical practice.^5,6^ Older patients, women, and patients in racial/ethnic minority groups are often underrepresented in clinical trials.^7–10^ Observational clinical and comparative effectiveness research thus has the potential to better inform clinical decision making, especially where gaps exist in evidence from clinical trials.^11^

To date, a few observational study analyses have explored the association of application of guideline-recommended therapies with clinical outcomes in HF,^12–15^ but not the specific and incremental contribution of each therapy. We sought to explore the individual and incremental gains associated with each of 7 current guideline-recommended therapies for HF and to evaluate their association with 24-month survival in outpatients with chronic HF and reduced LVEF. To accomplish this, we conducted a nested case-control study of patients with HF drawn from the Registry to Improve the Use of Evidence-Based Heart Failure Therapies in the Outpatient Setting (IMPROVE HF) cohort.

## Methods

### Study Population

The study population was drawn from the longitudinal cohort of the IMPROVE HF registry. IMPROVE HF was a prospective study designed to evaluate the effectiveness of a practice-specific performance improvement intervention on the use of selected guideline-recommended therapies for outpatients with diagnosed HF and reduced LVEF or prior myocardial infarction and LVEF. The overall study objectives and methods were reported previously in detail.^16,17^ Community and academic single- and multispecialty cardiology outpatient practices from across the United States were invited to participate. Patients with a clinical diagnosis of HF or prior myocardial infarction documented on at least 2 separate visits were eligible for enrollment. Reduced LVEF was required to be demonstrated by a quantitative LVEF ≤35% or by qualitative findings of moderate to severe left ventricular systolic dysfunction on the most recent echocardiogram, nuclear multiple gated acquisition scan, contrast ventriculogram, or magnetic resonance imaging scan. Patients with a noncardiovascular medical condition with an estimated survival of <1 year and those who had undergone cardiac transplantation were excluded.

### Data Collection

Data were collected by medical chart review.^16,17^ Use of selected guideline-recommended therapies was assessed at baseline at all participating practices. After baseline data collection, a practice-specific performance improvement intervention was implemented, and use of recommended therapies was reassessed at 12 and 24 months at all practices. Patient demographic and clinical characteristics were also collected, as were medical history, previous treatments, New York Heart Association (NYHA) functional status, QRS duration, laboratory and diagnostic tests and results, treatments, and provision of HF education. Contraindications and other reasons for not administering the recommended therapies (eg, patient noncompliance, patient refusal, and medical, economic, social, and religious reasons) were collected from the chart when documented. Collected practice characteristics included geographic region, outpatient practice setting (ie, university or teaching versus nonuniversity or nonteaching), practice type (single versus multispecialty), presence of a HF clinic in the practice, staffing (ie, number of physicians, HF nurses, electrophysiologists, and interventionalists in the practice), number of patients managed annually, type of medical record system (electronic, paper, or mixed), and use of IMPROVE HF performance improvement tools.

For each practice, medical records from patients with a clinical diagnosis of HF or prior myocardial infarction and LVEF ≤35% were screened, and a representative sample of 90 patients per practice (on average) was randomly selected for each follow-up assessment.^16,17^ The study was designed with several measures to ensure data quality and accuracy. These measures included the use of 34 trained, centralized chart reviewers, who received ongoing training and testing by members of the IMPROVE HF Steering Committee to ensure the accuracy of data abstraction.^16^ Average interrater reliability (κ statistic) was 0.82.^17^ Data quality reports were generated monthly, and an average of 1.7 automated data quality checks were performed on each data field to ensure the values met prespecified ranges, formats, and units. An audit of all patient data as compared with source documentation was conducted for 20% of the entire patient sample, using records from a 10% random sample of practices. The mean data concordance rate was 94.5% (range, 92.3% to 96.3%).^17^ Practices were required to obtain institutional review board approval or waivers to participate in IMPROVE HF.^16^ The registry coordinating center was Outcome Sciences, Inc (Cambridge, MA).

### Definition of Cases and Controls

A nested matched case-control design was used because a large cohort of patients was available in the IMPROVE-HF registry, enabling more explicit control of known powerful confounders, and because such an analysis would be less impacted by loss to follow-up. Cases were defined as patients with HF who died from any cause within 24 months of follow-up. All-cause mortality was used because it is less subject to interpretation and was collected in IMPROVE HF. Cohort patients who survived to 24 months were considered eligible controls.

### Matching

Cases and controls were matched on the basis of their propensity score and matched at a 1:2 ratio using the greedy matching technique.^18^ The propensity score in this analysis was the probability of death. A logistic regression model was used to generate the probability of death with dead/live as the outcome and the following baseline characteristics as covariates: Patient characteristics in the model were age, race, insurance status, HF etiology, atrial fibrillation, diabetes, chronic obstructive pulmonary disease, coronary artery bypass graft, peripheral vascular disease, depression, New York Heart Association functional status, rales, edema, LVEF, systolic blood pressure, diastolic blood pressure, heart rate, sodium, blood urea nitrogen, creatinine, potassium, and QRS duration; practice characteristics in the model were number of electrophysiologists in practice, number of cardiologists in practice, type of medical record system, and outpatient practice setting. No imputation was done for any of the missing values, so the match was generated on the basis of the nonmissing covariates. Variables with a large proportion of missing values, such as type B natriuretic peptide and ethnicity, were not used for the match.

### Guideline-Recommended Therapies

Seven guideline-recommended therapies were selected during trial design by the IMPROVE HF Steering Committee^16^: use of (1) angiotensin-converting enzyme inhibitor or angiotensin receptor blocker (ACEI/ARB), (2) β-blocker, (3) aldosterone antagonist, (4) anticoagulation for atrial fibrillation/flutter, (5) cardiac resynchronization therapy (CRT) with a pacemaker or defibrillator, (6) implantable cardioverter-defibrillator (ICD or CRT with defibrillator), and (7) patient education about HF. Each therapy was selected on the basis of its potential to improve patient outcomes, precision of definition, construct and content validity, and feasibility.^16^ Patients who met the guideline-specified eligibility criteria for each individual therapy, with no contraindications, intolerance, or other documented reasons for not receiving it, were eligible for inclusion in the analyses for that measure.^16,17^ Use of the HF education measure was based on documentation in the medical record that the patient had received written or verbal education about HF. Documentation of New York Heart Association functional class at a level consistent with guideline specifications is required to be eligible for aldosterone antagonist, CRT, or ICD therapy; thus, only patients with quantitative or qualitative evidence of New York Heart Association functional class documented in the medical record were included in analyses for those measures.

### Statistical Analysis

The IMPROVE HF longitudinal cohort consisted of 15 177 patients evaluated at the baseline, 12-month, and 24-month assessments. For this study, the data collection forms were required to have complete baseline data and vital status recorded at 24 months. Descriptive summary statistics of baseline patient and practice characteristics were calculated for the case and control groups and both groups combined. Continuous variables were analyzed using the 2-sample *t* test, and categorical variables were analyzed with the chi-square test. The baseline treatment rate for each of the 7 quality measures also was calculated for the case and control groups, and differences between groups were compared using the chi-square test.

The primary analysis was designed to evaluate the association between baseline use of the 7 guideline-recommended HF therapies and mortality within 24 months of follow-up. For each HF therapy, for eligible patients, the unadjusted odds ratio (OR) of death was determined using a logistic regression model with the therapy as the predictor variable and no covariate adjustment. A univariate logistic regression analysis was then performed for each patient and practice characteristic assessed in this study to identify potential covariates for the multivariate logistic model. All characteristics with a *P* value ≤0.10 in the univariate regression were considered potential confounders. These characteristics were fitted into a multivariate logistic regression model, with treatment as the main effect and the potential confounders as covariates, to determine the OR of death for each guideline-recommended therapy among therapy-eligible patients who received the treatment at baseline versus therapy-eligible patients who did not receive baseline treatment in each study group. Therapies were sequenced on the basis of their β-coefficients and the order in which they are commonly prescribed in clinical practice. Patients who were not treated at baseline but who crossed over to treatment by month 12 of the study were considered as treated at baseline. Sensitivity analyses with these early crossover patients considered as untreated were also performed.

An additional set of analyses was conducted to evaluate the association between total number of guideline-recommended therapies received by all patients at baseline and death within 24 months. A logistic regression analysis using dead versus alive as the outcome, number of therapies as the main effect, and adjusting for other covariates, was conducted to calculate ORs and 95% confidence intervals (CIs) for each comparison group. Patients who received 0 or 1 guideline-recommended therapy were grouped together, as were those who received 5, 6, or 7 therapies, because of the low number of patients receiving 0, 6, or 7 therapies. Reference value 0/1 was used and was compared with 2, 3, 4, and 5/6/7 therapies. To assess the cumulative contribution of applying each guideline-recommended therapy, we used a logistic regression model to calculate the OR of death at 24 months for each therapy among the entire case-control population irrespective of eligibility. The estimated cumulative contribution of applying each of the guideline-recommended therapies sequentially was calculated by adding the β-coefficients for each therapy in order of greatest to least impact, as presented by the OR of death for multiple treatments versus no treatment. We also performed an analysis of the sequential contribution of ACEI/ARB followed by β-blockers, followed by CRT plus ICD, confined to the subgroup of patients eligible for each of these 4 key guideline-directed HF therapies. *P*<0.05 was considered statistically significant for all analyses unless otherwise noted. Analyses were completed with SAS statistical software, version 9.1 (SAS Institute, Cary, NC).

All authors had full access to the data and take responsibility for its integrity. All authors approved the manuscript as written.

## Results

Of the 15 177 patients in the IMPROVE HF longitudinal cohort, 4128 (27.2%) were selected through the propensity score matching process. Of these, 1376 had a vital status of dead at 24 months (cases), and 2752 were alive at 24 months (controls). Overall, patient characteristics were very well matched between groups (Table 1). Mean (standard deviation) age was 72.0 years (12.4 years) and 71.6 years (11.5 years) in cases and controls, respectively (*P*=0.2396), and 71.2% and 72.2%, respectively, were male (*P*=0.5246). Race was the only statistically significantly different characteristic between cases and controls (*P*=0.0006). Baseline practice characteristics, calculated at the patient level, were also very well matched between the case and control groups (Table 2). The majority of practices in both groups were not affiliated with a university or teaching facility (68.5% and 68.2% for cases and controls, respectively), and the median number of patients managed annually was 2319. Practices were also well matched in terms of the number of cardiologists, electrophysiologists, interventionalists, and HF nurses on staff.

**Table 1:** Baseline Patient Characteristics for the 1:2 Matched Cohort

	Cohort			
		
Characteristic	Total (N=4128)	Case (Dead) (N=1376)	Control (Alive) (N=2752)	*P*
Age, mean (SD), y	71.7 (11.8)	72.0 (12.4)	71.6 (11.5)	0.2396

Sex, *n* (%)				0.5246

Male	2966 (71.9%)	980 (71.2%)	1986 (72.2%)	

Female	1162 (28.1%)	396 (28.8%)	766 (27.8%)	

Race, *n* (%)				0.0006

Black	377 (9.1%)	145 (10.5%)	232 (8.4%)	

White	1869 (45.3%)	627 (45.6%)	1242 (45.1%)	

Other	69 (1.7%)	14 (1.0%)	55 (2.0%)	

Not documented/missing	1813 (43.9%)	590 (42.9%)	1223 (44.4%)	

Insurance, *n* (%)				0.4963

Medicare	2829 (68.5%)	953 (69.3%)	1876 (68.2%)	

Medicaid	134 (3.2%)	52 (3.8%)	82 (3.0%)	

Private	786 (19.0%)	242 (17.6%)	544 (19.8%)	

Other	118 (2.9%)	43 (3.1%)	75 (2.7%)	

None	37 (0.9%)	12 (0.9%)	25 (0.9%)	

Not documented/missing	224 (5.4%)	74 (5.4%)	150 (5.5%)	

Ischemic HF etiology, *n* (%)	2893 (70.1%)	996 (72.6%)	1897 (69.3%)	0.0781

History of atrial fibrillation	1474 (35.7%)	492 (35.8%)	982 (35.7%)	0.9634

History of diabetes	1464 (35.5%)	495 (36%)	969 (35.2%)	0.6290

History of hypertension	2612 (63.3%)	874 (63.5%)	1738 (63.2%)	0.8194

Previous MI	1715 (41.5%)	557 (40.5%)	1158 (42.1%)	0.3258

History of COPD	801 (19.4%)	285 (20.7%)	516 (18.8%)	0.1329

History of coronary artery bypass grafting	1413 (34.2%)	476 (34.6%)	937 (34.0%)	0.7279

History of PCI	1082 (26.2%)	361 (26.2%)	721 (26.2%)	0.9800

History of PVD	514 (12.5%)	176 (12.8%)	338 (12.3%)	0.6407

History of depression	410 (9.9%)	151 (11.0%)	259 (9.4%)	0.1136

NYHA class, *n* (%)				0.2379

I	1335 (32.3%)	419 (30.5%)	916 (33.3%)	

II	1612 (39.1%)	558 (40.6%)	1054 (38.3%)	

III	990 (24%)	331 (24.1%)	659 (23.9%)	

IV	84 (2%)	34 (2.5%)	50 (1.8%)	

Not documented	107 (2.6%)	34 (2.5%)	73 (2.7%)	

LVEF, %				0.3102

Mean (SD)	25.1 (7)	24.9 (7)	25.2 (7)	

Median (25th, 75th percentiles)	25 (20, 30)	25 (20, 30)	25 (20, 30)	

Systolic blood pressure, mm Hg, median (25th, 75th percentiles)	118 (106, 130)	118 (106, 130)	118 (108, 130)	0.7506

Diastolic blood pressure, mm Hg, median (25th, 75th percentiles)	70 (60, 76)	69 (60, 76)	70 (60, 76)	0.0785

Resting heart rate, beats/min, median (25th, 75th percentiles)	72 (64, 80)	72 (64, 80)	71.5 (64, 79)	0.7007

Pulmonary rales on last examination, *n* (%)	144 (3.5%)	41 (3.0%)	103 (3.7%)	0.4515

Edema on last examination, *n* (%)	896 (21.7%)	313 (22.7%)	583 (21.2%)	0.5173

Sodium, mEq/L, median (25th, 75th percentiles)	140 (137, 142)	139 (137, 142)	140 (137, 142)	0.5941

Serum urea nitrogen, mg/dL, median (25th, 75th percentiles)	23 (17, 31)	23 (17, 32)	23 (17, 30.5)	0.3465

Creatinine, mg/dL, median (25th, 75th percentiles)	1.3 (1, 1.6)	1.3 (1, 1.6)	1.3 (1, 1.6)	0.1723

Potassium, mEq/L, median (25th, 75th percentiles)	4.4 (4.1, 4.7)	4.4 (4.1, 4.8)	4.4 (4.1, 4.7)	0.8566

QRS duration, ms, median (25th, 75th percentiles)	130 (102, 160)	126 (104, 158)	132 (102, 160)	0.7010

COPD indicates chronic obstructive pulmonary disease; HF, heart failure; LVEF, left ventricular ejection fraction; MI, myocardial infarction; NYHA, New York Heart Association; PCI, percutaneous coronary intervention; PVD, peripheral vascular disease; and SD, standard deviation.

**Table 2.** Baseline Practice Characteristics: Patient-Level Analysis

	Cohort			
		
Characteristic	Total (N=4128)	Case (Dead) (N=1376)	Control (Alive) (N=2752)	*P*
Census region, *n* (%)				0.6075

South	1613 (39.1%)	553 (40.2%)	1060 (38.5%)	

West	584 (14.1%)	185 (13.4%)	399 (14.5%)	

Central	800 (19.4%)	258 (18.8%)	542 (19.7%)	

Northeast	1131 (27.4%)	380 (27.6%)	751 (27.3%)	

Outpatient practice setting, *n* (%)				0.2360

Nonuniversity, nonteaching	2821 (68.3%)	943 (68.5%)	1878 (68.2%)	

Nonuniversity, teaching	914 (22.1%)	316 (23%)	598 (21.7%)	

University, teaching	393 (9.5%)	117 (8.5%)	276 (10%)	

Multispecialty, *n* (%)	943 (22.8%)	325 (23.6%)	618 (22.5%)	0.4015

Electronic health record system, *n* (%)				0.3758

Paper	1933 (46.8%)	636 (46.2%)	1297 (47.1%)	

Electronic	1443 (35.0%)	473 (34.4%)	970 (35.2%)	

Mixed	752 (18.2%)	267 (19.4%)	485 (17.6%)	

HF nurse in practice (>1 FTE APN), *n* (%)	1698 (41.1%)	553 (40.2%)	1145 (41.6%)	0.3628

No. of electrophysiologists in practice				0.4108

Mean (SD)	1.7 (1.7)	1.7 (1.7)	1.7 (1.8)	

Median (25th, 75th percentiles)	1 (0, 3)	1 (0, 3)	1 (0, 3)	

No. of interventionalists in practice				0.6872

Mean (SD)	5.1 (3.3)	5 (3.3)	5.1 (3.3)	

Median (25th, 75th percentiles)	5 (3, 6)	5 (3, 6)	5 (3, 6)	

No. of HF clinic sessions in practice				0.9649

Mean (SD)	1.5 (0.5)	1.5 (0.5)	1.5 (0.5)	

Median (25th, 75th percentiles)	2 (1, 2)	2 (1, 2)	2 (1, 2)	

No. of cardiologists in practice				0.2364

Mean (SD)	13.9 (11.6)	13.6 (10.9)	14 (11.9)	

Median (25th, 75th percentiles)	10 (7, 18)	10 (7, 18.5)	10 (7, 18)	

No. of HF patients managed annually				0.6948

Mean (SD)	3601 (3968.6)	3565 (3820.2)	3619.4 (4043)	

Median (25th, 75th percentiles)	2319 (700, 5000)	2319 (750, 5000)	2319 (700, 5000)	

APN indicates advanced practice nurse; FTE, full-time equivalent; HF, heart failure; SD, standard deviation.

**Figure 1.**
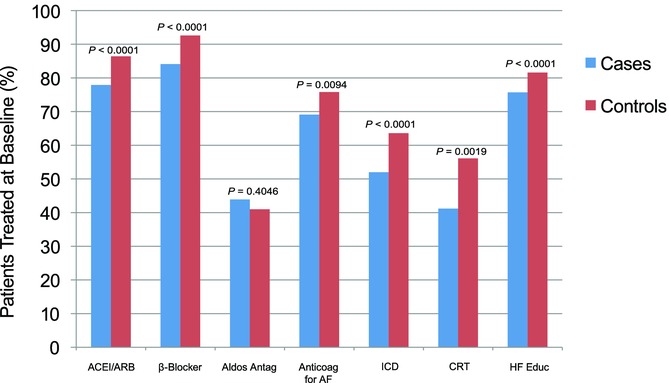
Use of guideline-recommended therapies at baseline in cases and controls. Baseline use of each of the guideline-recommended therapies for cases (dead at 24 mo) compared with controls (alive at 24 mo). ACEI indicates angiotensin-converting enzyme inhibitor; AF, atrial fibrillation; ARB, angiotensin receptor blocker; CRT, cardiac resynchronization therapy (with defibrillator or pacemaker); HF, heart failure; ICD, implantable cardioverter-defibrillator (including CRT with defibrillator).

Figure 1 shows the proportion of patients in the case and control groups who received therapies for which they were eligible at baseline. Baseline treatment rates were significantly higher among controls who were eligible for an ACEI/ARB, β-blocker, anticoagulation for atrial fibrillation, ICD, CRT, and HF education than among eligible cases. There was no difference between the groups for patients eligible for aldosterone antagonists. The baseline treatment rates among patients in this nested analysis were similar to the rates observed in the overall IMPROVE HF patient population at baseline.^17^ The number and proportion of cases and controls newly initiated on therapy within 12 months are shown in Table 3.

**Table 3.** Proportion of Treated Patients Who Received Treatment at Baseline and Proportion Who Crossed Over to Treatment by 12 Months*

Therapy	Treatment at Baseline	No Treatment at Baseline, Crossed Over to Treatment by 12 Months
ACEI/ARB		

Cases (dead)	936 (96.6%)	33 (3.4%)

Controls (alive)	2062 (92.8%)	159 (7.2%)

β-Blocker		

Cases (dead)	1018 (96.1%)	41 (3.9%)

Controls (alive)	2206 (94.6%)	126 (5.4%)

Aldosterone antagonists		

Cases (dead)	118 (92.9%)	9 (7.1%)

Controls (alive)	203 (82.5%)	43 (17.5%)

Anticoagulation for atrial fibrillation		

Cases (dead)	292 (95.1%)	15 (4.9%)

Controls (alive)	652 (94.4%)	39 (5.6%)

ICD		

Cases (dead)	453 (88.3%)	60 (11.7%)

Controls (alive)	1016 (81.9%)	224 (18.1%)

CRT		

Cases (dead)	49 (72.1%)	19 (27.9%)

Controls (alive)	121 (67.2%)	59 (32.8%)

HF education		

Cases (dead)	874 (83.9%)	168 (16.1%)

Controls (alive)	1688 (75.2%)	558 (24.8%)

ACEI indicates angiotensin-converting enzyme inhibitor; ARB, angiotensin receptor blocker; CRT, cardiac resynchronization therapy; HF, heart failure; ICD, implantable cardioverter-defibrillator.

* This table shows the proportion of those patients treated at baseline of the total patients treated and the proportion of patients with early crossover of the total patients treated.

Table 4 shows the unadjusted and adjusted ORs for 24-month mortality among patients eligible for each therapy at baseline. After adjustment for patient and practice characteristics, all guideline-recommended therapies except aldosterone antagonists were shown to be independently associated with a lower odds of death at 24 months. Use of β-blockers and CRT were associated with the greatest reductions in the odds of death (β-blocker 58% lower adjusted odds, adjusted OR 0.42, 95% CI, 0.34–0.52, *P*<0.0001 and CRT 56% lower adjusted odds, adjusted OR 0.44, 95% CI, 0.29–0.67, *P*=0.0001). ACEI/ARB, ICD, HF education, and anticoagulation for atrial fibrillation were also independently associated with lower adjusted odds of death. There was no significant difference in the adjusted odds of death associated with use compared with nonuse of aldosterone antagonists. In the sensitivity analyses, use of β-blockers, ACEI/ARB, CRT, and ICD was also associated with statistically significantly reduced odds of death at 24 months.

**Table 4.** Association Between Treatment With Guideline-Recommended Therapy at Baseline and Mortality Within 24 Months of Follow-Up: Univariate and Multivariate Logistic Regression Models*

Therapy	Treated n (%)	Not Treated n (%)	Unadjusted OR (95% CI)	*P*	Adjusted OR (95% CI)	*P*
ACEI/ARB						

Cases (dead)	969 (77.9%)	275 (22.1%)	0.56 (0.47–0.66)	<0.0001	0.56 (0.47–0.67)	<0.0001

Controls (alive)	2221 (86.4%)	351 (13.6%)				

β-Blocker						

Cases (dead)	1059 (84.1%)	200 (15.9%)	0.42 (0.34–0.52)	<0.0001	0.42 (0.34–0.52)	<0.0001

Controls (alive)	2332 (92.6%)	185 (7.4%)				

Aldosterone antagonists						

Cases (dead)	127 (43.9%)	162 (56.1%)	1.13 (0.85–1.50)	0.4047	1.05 (0.74–1.51)	0.7707

Controls (alive)	246 (41.0%)	354 (59.0%)				

Anticoagulation for atrial fibrillation						

Cases (dead)	307 (69.1%)	137 (30.9%)	0.72 (0.56–0.92)	0.0096	0.73 (0.57–0.95)	0.0179

Controls (alive)	691 (75.8%)	221 (24.2%)				

ICD						

Cases (dead)	513 (52.0%)	473 (48.0%)	0.62 (0.53–0.72)	<0.0001	0.62 (0.53–0.73)	<0.0001

Controls (alive)	1240 (63.6%)	709 (36.4%)				

CRT						

Cases (dead)	68 (41.2%)	97 (58.8%)	0.55 (0.38–0.80)	0.0020	0.44 (0.29–0.67)	0.0001

Controls (alive)	180 (56.1%)	141 (43.9%)				

HF education						

Cases (dead)	1042 (75.7%)	334 (24.3%)	0.70 (0.60–0.82)	<0.0001	0.73 (0.62–0.85)	<0.0001

Controls (alive)	2246 (81.6%)	506 (18.4%)				

ACEI indicates angiotensin-converting enzyme inhibitor; ARB, angiotensin receptor blocker; COPD, chronic obstructive pulmonary disease; CRT, cardiac resynchronization therapy; DBP, diastolic blood pressure; EHR, electronic health record; HF, heart failure; ICD, implantable cardioverter-defibrillator; LVEF, left ventricular ejection fraction; NYHA, New York Heart Association; SBP, systolic blood pressure.

* The analysis for each individual therapy included only patients eligible for that therapy. Variables retained in the models were as follows: ACEI/ARB: race, depression, DBP; β-blocker: race, HF etiology, DBP; aldosterone antagonist: age, sex, COPD, LVEF, SBP, DBP, QRS, HF clinic in practice, EHR group; anticoagulation for atrial fibrillation: age, NYHA class, DBP, multispecialty practice; ICD: EHR group; CRT: age, edema, LVEF, SBP, DBP, HF clinic in practice, EHR group, APN in practice, outpatient setting; and HF education: race, HF etiology, DBP.

**Figure 2.**
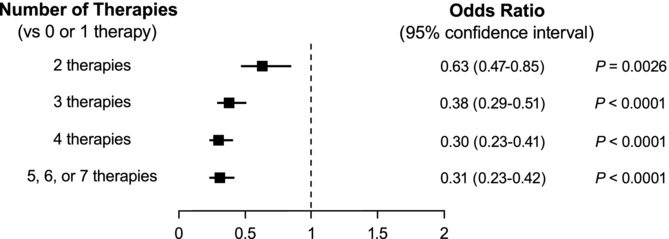
ORs for 24-month mortality associated with the number of guideline-recommended therapies received at baseline. Analysis includes all patients from the case-control population (*N*=4128). The number (%) of patients receiving each number of therapies at baseline was as follows: 0 or 1, 238 (5.8%); 2, 712 (17.3%); 3, 1327 (32.2%); 4, 1123 (27.2%); and 5, 6, or 7, 728 (17.6%). OR indicates odds ratio.

**Figure 3.**
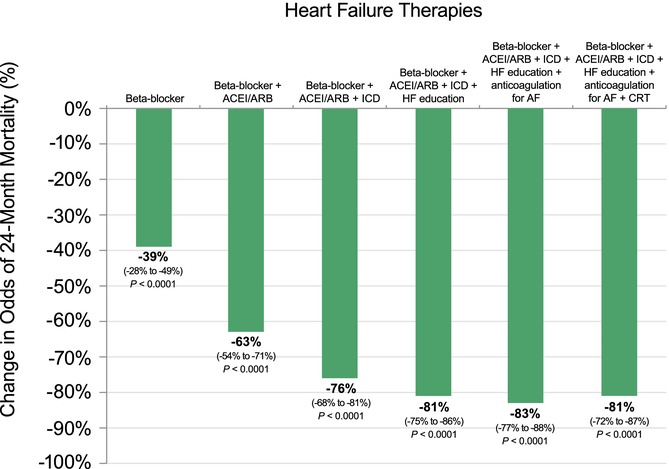
Cumulative percent reduction in odds of death at 24 months with each sequentially applied guideline-recommended HF therapy. Therapies were sequenced on the basis of their β-coefficients and the order in which they are commonly applied clinically. Variables retained in the model were race, HF etiology, and diastolic blood pressure. Incremental *P* values for the sequentially applied therapies (left to right) were as follows: <0.0001, <0.0001, <0.0001, 0.0038, 0.1388, and 0.1208, respectively. ACEI indicates angiotensin-converting enzyme inhibitor; AF, atrial fibrillation; ARB, angiotensin receptor blocker; CRT, cardiac resynchronization therapy (with defibrillator or pacemaker); HF, heart failure; ICD, implantable cardioverter-defibrillator (including CRT with defibrillator).

**Figure 4.**
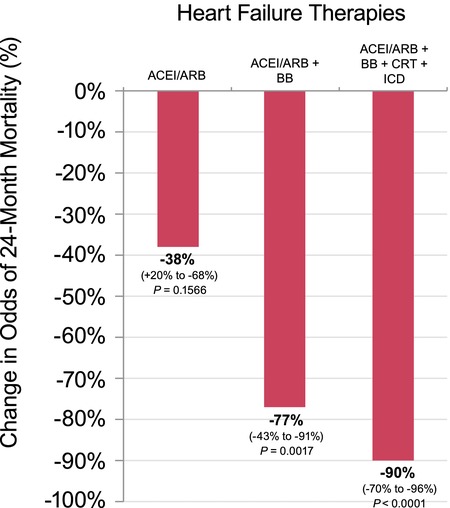
Cumulative percent reduction in odds of death at 24 months associated with sequential treatments compared with no treatment. Analysis includes only patients eligible for all 4 therapies (*N*=368). ACEI indicates angiotensin-converting enzyme inhibitor; ARB, angiotensin receptor blocker; CRT, cardiac resynchronization therapy; ICD, implantable cardioverter-defibrillator.

Incremental benefits for the guideline-recommended therapies were observed. Patients who received a greater number of treatments at baseline were more likely to be alive at 24 months (Figure 2). The benefit tended to plateau after 4 to 5 therapies. Sequential application of specific therapies, in order of greatest to least individual associated benefit (β-Blocker, ACEI/ARB, ICD, HF education, and anticoagulation for atrial fibrillation), yielded the lowest odds of death at 24 months (Table 5 and Figure 3). Treatment with these 5 therapies, as compared with no treatment, was independently associated with an 83% reduction in the odds of death within 24 months (adjusted OR 0.17, 95% CI, 0.12–0.23, *P*<0.0001). When we separately analyzed whether ACEI/ARB, β-blockers, CRT, and ICD would provide incremental benefit with each successive therapy if added to the model in the order in which they are generally applied in clinical practice, including only those patients eligible for all 4 therapies (*N*=368), successive benefit was found for ACEI/ARB, ACEI/ARB+β-blocker, and ACEI/ARB+β-blocker+CRT+ICD compared with no treatment (all 4 treatments compared with 0 treatments, adjusted OR 0.10, 95% CI, 0.04–0.30, *P*<0.0001; Figure 4).

**Table 5.** Cumulative Effect of Sequential Application of Guideline-Recommended Heart Failure Therapies on 24-Month Mortality*

Therapy	No. (%) of Patients	Adjusted OR (95% CI)	*P*	*P* (incremental)
β-Blocker	3477 (84.2%)	0.61 (0.51–0.72)	<0.0001	<0.0001

β-Blocker+ACEI/ARB	2461 (59.6%)	0.37 (0.29–0.46)	<0.0001	<0.0001

β-Blocker+ACEI/ARB+ICD	1397 (33.8%)	0.24 (0.19–0.32)	<0.0001	<0.0001

β-Blocker+ACEI/ARB+ICD+HF education	1169 (28.3%)	0.19 (0.14–0.25)	<0.0001	0.0038

β-Blocker+ACEI/ARB+ICD+HF education+anticoagulation for AF	400 (9.7%)	0.17 (0.12–0.23)	<0.0001	0.1388

β-Blocker+ACEI/ARB+ICD+HF education+anticoagulation for AF+CRT	81 (2.0%)	0.19 (0.13–0.28)	<0.0001	0.1208

ACEI indicates angiotensin-converting enzyme inhibitor; AF, atrial fibrillation; ARB, angiotensin receptor blocker; CI, confidence interval; CRT, cardiac resynchronization therapy (with defibrillator or pacemaker); HF, heart failure; ICD, implantable cardioverter-defibrillator (including CRT with defibrillator); OR, odds ratio.

* Variables retained in the model were race, HF etiology, and diastolic blood pressure.

## Discussion

This study is among the first to examine the individual and incremental clinical effectiveness of guideline-recommended therapies for patients with HF and reduced LVEF. Analysis of IMPROVE HF data using a nested case-control approach revealed that with one exception, each of these guideline-recommended therapies was associated with a decreased risk of 24-month mortality. β-Blocker and CRT therapy had the strongest 24-month survival benefits observed. In addition, there was incremental benefit with each successive guideline-recommended therapy. This strong positive association between progressive use of guideline-recommended therapies and improved risk-adjusted survival did appear to plateau after any 4 to 5 therapies were applied. These findings provide further evidence supporting the clinical effectiveness of guideline-recommended HF therapies for patients encountered in real-world clinical practice and suggest that there may be incremental benefits to the application of these therapies in the outpatient practice setting. These data may also provide further rationale for using systems, performance improvement, and disease management programs to ensure the implementation of guideline-recommended HF therapies into clinical practice.

The benefits associated with pharmacological and device therapies observed in this study are consistent with key clinical trials in HF. The findings of strong associations between β-blocker and ACEI/ARB use and improved survival are consistent with the wealth of clinical trial data demonstrating the efficacy of these therapies and with prior studies of clinical effectiveness of these agents.^14,19–21^ A study of Medicare patients with HF and left ventricular systolic dysfunction showed that a discharge prescription for either an ACEI or ARB was associated with a 17% relative reduction in 1-year postdischarge mortality after risk adjustment.^21^ A clinical effectiveness study of β-blocker therapy among Medicare beneficiaries hospitalized with HF found that the use of β-blocker therapy was independently associated with improved survival.^14^ The current IMPROVE HF study provides further evidence supporting the clinical effectiveness of ACEI/ARB and β-blocker use and extends these findings to a cohort not entirely limited to Medicare patients. In the present analysis, anticoagulation for atrial fibrillation was also associated with a reduced risk of mortality. This finding is consistent with clinical trials demonstrating that anticoagulation for atrial fibrillation reduces the risk of fatal and nonfatal stroke in patients with HF.^22^ The clinical effectiveness of CRT and ICD therapy is also supported by this study. Use of CRT and ICD therapies has been shown to reduce mortality in randomized clinical trials.^15,23^ In our study, CRT was strongly and independently associated with improved patient survival at 24 months. CRT has previously been shown to be clinically effective in patients with HF.^23^ In the present study, after adjustment for covariates, ICD use was significantly associated with a 38% lower odds of 2-year mortality. Prior studies have also shown that ICD use in eligible patients at the time of hospital discharge is associated with reduced mortality over 1- and 3-year periods.^14,15^ In contrast, aldosterone antagonist use was not associated with lower mortality after multivariable adjustment. These findings are in contrast to randomized clinical trials demonstrating efficacy^1^ and prior studies of aldosterone antagonist use being associated with lower mortality risk.^14^ These findings may have resulted from confounding by indication or other forms of observational bias. Alternatively, aldosterone antagonist use may be less effective or less safe as dosed and monitored in clinical practice.^9^ Further studies of the clinical effectiveness of aldosterone antagonists in HF in diverse clinical practice settings are needed.

As previously noted, by the very nature of placebo-controlled randomized clinical trial design, new evidence supporting new therapies provides evidence that is considered additive.^24^ Hence, these studies inform clinicians about the treatments that should be prescribed but rarely provide information about which therapies may be omitted. Studies weighing the incremental value of well-established therapies in the modern era of HF treatment have not been performed.^24^ Thus, the evidence of incremental benefit provided by guideline-recommended HF therapies evaluated in the outpatient setting for patients with chronic HF is valuable. Using a nested case-control design, we were able to weigh the impact of several guideline-recommended HF therapies as applied in current clinical practice in real-world patients. With this analysis, estimates of the survival advantage associated with some of these therapies are in some cases greater than those observed in clinical trials. This may be because patients enrolled in randomized trials may not be fully representative of HF patients in clinical practice. It is possible that this latter group may derive even greater benefit from the use of guideline-recommended therapies. Nevertheless, selection bias and residual confounding may also account for these findings.

With the expanding evidence base provided by clinical trials, the number of evidence-based, guideline-recommended HF therapies has increased. This increase has been considered by some to place additional burdens on patients and physicians in terms of adherence and on health systems in terms of resource allocation. By weighing the benefits of each guideline-recommend therapy with respect to 24-month survival, this analysis may inform the choice between therapies and strategies when such decisions need to be made. In addition, this analysis explored the relative mortality reduction associated with each guideline-recommended therapy and describes the proportion of lives that may be preserved with more complete application of each of the HF therapies. At a clinical level, being able to independently value these HF therapies may provide the rationale for choosing among treatments when a choice must be made, whether for reasons of cost, tolerance, or adherence. Among the HF therapies evaluated in this analysis, β-blocker and CRT seemed to impart the greatest individual benefits. These therapies may be among the priorities for focused efforts to improve implementation of HF quality improvement programs such as Get With The Guidelines-HF. The finding of a potential plateau in survival benefit after any 4 to 5 guideline-recommended HF therapies were applied, if confirmed in additional studies, may also help to better inform clinical decision making and the design of HF quality improvement efforts.

### Limitations

Certain limitations inherent in the design of IMPROVE HF and this analysis should be considered. Medical chart review with data abstraction was the source of patient clinical data. Every effort was made to ensure the accuracy and completeness of these data through consistent regular training of personnel involved in the medical chart review process, but it is possible that errors and omissions could have occurred. It is also possible that some proportion of patients considered eligible for treatment who were not treated at each time point may have had contraindications or other reasons that prevented treatment but were not documented in the medical record. In addition, this analysis was confined to patients with complete follow-up at 24 months, and in the primary analyses patients with early crossover to treatment were considered as treated at baseline, which may have introduced bias. As with all observational studies, the possibility for unmeasured bias exists, thus, leading to overestimation or underestimation of treatment effects. We could not adjust for socioeconomic factors or patient adherence. With the case-control design, the individual therapy analyses did not adjust for use of other background therapies. There are differential indications for each therapy as a function of HF severity, which, even after propensity matching and risk adjustment, may still have influenced the incremental benefit analyses. There may also be other measured or unmeasured confounding variables that would have strengthened or weakened the association for some or all of the therapies. The majority of patients who received CRT received a CRT-defibrillator device. This high concordance for these two therapies likely diminished the ability to ascertain the incremental benefit of CRT in some of the analyses. The associations between use of guideline-recommended therapies and mortality do not determine causality. Although these associations may reflect the clinical effectiveness of these treatments, they may alternatively reflect treatment selection bias, which would tend to favor these associations. The ORs may have been magnified by confounding. Although the baseline treatment rates in IMPROVE HF were similar to those of other studies of HF patients in cardiology practices,^25^ these findings may not apply to practices that differ from the IMPROVE HF outpatient cardiology practices.

## Conclusions

This analysis of IMPROVE HF data demonstrates that guideline-recommended therapies for patients with HF and reduced LVEF are associated with decreased risk of 24-month mortality. β-Blocker and CRT therapy had the strongest 24-month survival benefits observed. In addition, there was an incremental benefit with each successive guideline-recommended therapy. This strong positive association between progressive use of guideline-recommended therapies and improved risk-adjusted survival plateaued after any 4 to 5 therapies were applied. These findings provide further evidence for the clinical effectiveness of guideline-recommended HF therapies among patients encountered in real-world clinical practice. Further, they suggest that benefit accrues incrementally with application of these therapies in the outpatient setting. These data provide further rationale for using systems, performance improvement, and HF disease management programs to ensure the implementation of guideline-recommended HF therapies into clinical practice.

## References

[1] Hunt SA, Abraham WT, Chin MH, Feldman AM, Francis GS, Ganiats TG, Jessup M, Konstam MA, Mancini DM, Michl K, Oates JA, Rahko PS, Silver MA, Stevenson LW, Yancy CW, Antman EM, Smith SC, Adams CD, Anderson JL, Faxon DP, Fuster V, Halperin JL, Hiratzka LF, Jacobs AK, Nishimura R, Ornato JP, Page RL, Riegel B (2005). ACC/AHA 2005 guideline update for the diagnosis and management of chronic heart failure in the adult: a report of the American College of Cardiology/American Heart Association Task Force on Practice Guidelines (Writing Committee to update the 2001 Guidelines for the Evaluation and Management of Heart Failure): developed in collaboration with the American College of Chest Physicians and the International Society for Heart and Lung Transplantation: endorsed by the Heart Rhythm Society. Circulation.

[2] Jessup M, Abraham WT, Casey DE, Feldman AM, Francis GS, Ganiats TG, Konstam MA, Mancini DM, Rahko PS, Silver MA, Stevenson LW, Yancy CW (2009). 2009 focused update: ACCF/AHA guidelines for the diagnosis and management of heart failure in adults: a report of the American College of Cardiology Foundation/American Heart Association Task Force on Practice Guidelines: developed in collaboration with the International Society for Heart and Lung Transplantation. Circulation.

[3] Lindenfeld J, Albert NM, Boehmer JP, Collins SP, Ezekowitz JA, Givertz MM, Katz SD, Klapholz M, Moser DK, Rogers JG, Starling RC, Stevenson WG, Tang WH, Teerlink JR, Walsh MN, Heart Failure Society of America (2010). HFSA 2010 comprehensive heart failure practice guideline. J Card Fail.

[4] Dickstein K, Cohen-Solal A, Filippatos G, McMurray JJ, Ponikowski P, Poole-Wilson PA, Strömberg A, van Veldhuisen DJ, Atar D, Hoes AW, Keren A, Mebazaa A, Nieminen M, Priori SG, Swedberg K, Vahanian A, Camm J, De Caterina R, Dean V, Dickstein K, Filippatos G, Funck-Brentano C, Hellemans I, Kristensen SD, McGregor K, Sechtem U, Silber S, Tendera M, Widimsky P, Zamorano JL, Task Force for Diagnosis and Treatment of Acute and Chronic Heart Failure 2008 of European Society of Cardiology, ESC Committee for Practice Guidelines (2008). ESC guidelines for the diagnosis and treatment of acute and chronic heart failure 2008: the Task Force for the Diagnosis and Treatment of Acute and Chronic Heart Failure 2008 of the European Society of Cardiology. Developed in collaboration with the Heart Failure Association of the ESC (HFA) and endorsed by the European Society of Intensive Care Medicine (ESICM). Eur Heart J.

[5] Committee on Comparative Effectiveness Research Prioritization, Institute of Medicine (2009). Initial National Priorities for Comparative Effectiveness Research.

[6] Lenfant C (2003). Shattuck lecture: clinical research to clinical practice—lost in translation?. N Engl J Med.

[7] Cherubini A, Oristrell J, Pla X, Ruggiero C, Ferretti R, Diestre G, Clarfield AM, Crome P, Hertogh C, Lesauskaite V, Prada GI, Szczerbinska K, Topinkova E, Sinclair-Cohen J, Edbrooke D, Mills GH (2011). The persistent exclusion of older patients from ongoing clinical trials regarding heart failure. Arch Intern Med.

[8] Masoudi FA, Havranek EP, Wolfe P, Gross CP, Rathore SS, Steiner JF, Ordin DL, Krumholz HM (2003). Most hospitalized older persons do not meet the enrollment criteria for clinical trials in heart failure. Am Heart J.

[9] Shah KB, Rao K, Sawyer R, Gottlieb SS (2005). The adequacy of laboratory monitoring in patients treated with spironolactone for congestive heart failure. J Am Coll Cardiol.

[10] Hernandez AF, Harrington RA (2008). Comparative effectiveness of angiotensin-converting-enzyme inhibitors: is an ACE always an ace?. CMAJ.

[11] Gibbons RJ, Gardner TJ, Anderson JL, Goldstein LB, Meltzer N, Weintraub WS, Yancy CW, American Heart Association Advocacy Coordinating Committee (2009). The American Heart Association's principles for comparative effectiveness research: a policy statement from the American Heart Association. Circulation.

[12] Fonarow GC, Albert NM, Curtis AB, Gheorghiade M, Heywood JT, Liu Y, Mehra MR, O'Connor CM, Reynolds D, Walsh MN, Yancy CW (2011). Associations between outpatient heart failure process-of-care measures and mortality. Circulation.

[13] Hernandez AF, Hammill BG, O'Connor CM, Schulman KA, Curtis LH, Fonarow GC (2009). Clinical effectiveness of beta-blockers in heart failure: findings from the OPTIMIZE-HF (Organized Program to Initiate Lifesaving Treatment in Hospitalized Patients with Heart Failure) registry. J Am Coll Cardiol.

[14] Hernandez AF, Hammill BG, Peterson ED, Yancy CW, Schulman KA, Curtis LH, Fonarow GC (2010). Relationships between emerging measures of heart failure processes of care and clinical outcomes. Am Heart J.

[15] Hernandez AF, Fonarow GC, Hammill BG, Al-Khatib SM, Yancy CW, O'Connor CM, Schulman KA, Peterson ED, Curtis LH (2010). Clinical effectiveness of implantable cardioverter-defibrillators among Medicare beneficiaries with heart failure. Circ Heart Fail.

[16] Fonarow GC, Yancy CW, Albert NM, Curtis AB, Stough WG, Gheorghiade M, Heywood JT, Mehra M, O'Connor CM, Reynolds D, Walsh MN (2007). Improving the use of evidence-based heart failure therapies in the outpatient setting: the IMPROVE HF performance improvement registry. Am Heart J.

[17] Fonarow GC, Albert NM, Curtis AB, Stough WG, Gheorghiade M, Heywood JT, McBride ML, Inge PJ, Mehra MR, O'Connor CM, Reynolds D, Walsh MN, Yancy CW (2010). Improving evidence-based care for heart failure in outpatient cardiology practices: primary results of the Registry to Improve the Use of Evidence-Based Heart Failure Therapies in the Outpatient Setting (IMPROVE HF). Circulation.

[18] Parsons LS (2004). Performing a 1:N case-control match on propensity score. http://www2.sas.com/proceedings/sugi29/toc.html#front.

[19] Fonarow GC, Abraham WT, Albert NM, Stough WG, Gheorghiade M, Greenberg BH, O'Connor CM, Pieper K, Sun JL, Yancy C, Young JB, OPTIMIZE-HF Investigators and Hospitals (2007). Association between performance measures and clinical outcomes for patients hospitalized with heart failure. JAMA.

[20] Patterson ME, Hernandez AF, Hammill BG, Fonarow GC, Peterson ED, Schulman KA, Curtis LH (2010). Process of care performance measures and long-term outcomes in patients hospitalized with heart failure. Med Care.

[21] Masoudi FA, Rathore SS, Wang Y, Havranek EP, Curtis JP, Foody JM, Krumholz HM (2004). National patterns of use and effectiveness of angiotensin-converting enzyme inhibitors in older patients with heart failure and left ventricular systolic dysfunction. Circulation.

[22] Shivkumar K, Jafri SM, Gheorghiade M (1996). Antithrombotic therapy in atrial fibrillation: a review of randomized trials with special reference to the Stroke Prevention in Atrial Fibrillation II (SPAF II) Trial. Prog Cardiovasc Dis.

[23] McAlister FA, Ezekowitz J, Hooton N, Vandermeer B, Spooner C, Dryden DM, Page RL, Hlatky MA, Rowe BH (2007). Cardiac resynchronization therapy for patients with left ventricular systolic dysfunction: a systematic review. JAMA.

[24] Chew DP, Anderson FA, Avezum Á, Eagle KA, FitzGerald G, Gore JM, Dedrick R, Brieger D, for the GRACE Investigators (2010). Six-month survival benefits associated with clinical guideline recommendations in acute coronary syndromes. Heart.

[25] Chan PS, Oetgen WJ, Buchanan D, Mitchell K, Fiocchi FF, Tang F, Jones PG, Breeding T, Thrutchley D, Rumsfeld JS, Spertus JA (2010). Cardiac performance measure compliance in outpatients: the American College of Cardiology and National Cardiovascular Data Registry's PINNACLE (Practice Innovation And Clinical Excellence) program. J Am Coll Cardiol.

